# Supplementary material for: Patterns of Intron Gain and Loss in Fungi
Source: PLoS Biol. 2004 Nov 30;2(12):e422. doi: 10.1371/journal.pbio.0020422 (PMC532390; doi:10.1371/journal.pbio.0020422)
Supplement: Table S1 — Also available at http://genes.mit.edu/NielsenEtAl/. (4.3 MB ZIP). [file pbio.0020422.st001.zip › NielsenEtAl/html/1403.html]

AN2244.1.NCU01183.1.MG08640.1.FG05139.1


```
 CLUSTAL W (1.82) Multiple Sequence Alignments - Introns Inserted


Sequence 1: NCU01183.1	372 aa
Sequence 2: MG08640.1	348 aa
Sequence 3: FG05139.1	371 aa
Sequence 4: AN2244.1	367 aa
Alignment Length: 372 aa
Number Identitical Residues: 282 aa
Alignment Score (without introns) 11709


MG08640.1 	MVNITEKIKE2----------~-----~--------~KLARLRAQLLEPGPGSGGGPGAG
NCU01183.1	MVNITEKIKE2IEEEMRKTQK1NKATE1YHLGLLKG2KLARLRAQLLEPGPGSGGGPGAG
FG05139.1 	MVNITEKIKE2IEEEMKRTQK1NKATE1YHLGLLKG2KLARLRAQLLEPGPGAGGGGGSG
AN2244.1  	MVNITEKIKE2IEDEMRRTQK1NKATE1YHLGLLKG2KLARLRAQLLEPTGGSGGG-GAG
          	**********  ...   :.. ..::.    .  .. ************  *:*** *:*

MG08640.1 	FDVSKSGDARISLVGFPSVGKSTFLSKVTKTRSEVAAYSFTTLTAIPGVLEYGGAEIQLL
NCU01183.1	FDVSKSGDARIALVGFPSVGKSTFLSKVTKTKSEVASYAFTTLTAIPGVLEYGGAEIQLL
FG05139.1 	FDVSKSGDARIALVGFPSVGKSTFLSKVTKTRSEVASYAFTTLTAIPGVLEYGGAEIQLL
AN2244.1  	FDVSKSGDARVALVGFPSVGKSTFLSKITKTKSEAAAYSFTTLTAIPGVLEYGGAEIQIL
          	**********::***************:***:**.*:*:*******************:*

MG08640.1 	DLPGIIEGAAEGKGRGRQVISAAK~TSDMILMVLDATKKAEQRALLEAELEAVGIRLNRE
NCU01183.1	DLPGIIEGASEGKGRGRQVISAAK~TSDLILMVLDATKRAEQRALLEAELEAVGIRLNRE
FG05139.1 	DLPGIIEGASEGKGRGRQVISAAK~TSDLILMVLDATKKAEQRALLEAELEAVGIRLNRE
AN2244.1  	DLPGIIEGASEGKGRGRQVISAAK0TSDLILMVLDATKKAEQRALLEAELDAVGIRLNKE
          	*********:************** ***:*********:***********:*******:*

MG08640.1 	PP~NIYLKPKKAGGMKINFASPPKR-LDEKMVYNILRDYKMLNAEVLIRDDNA~TVDDLI
NCU01183.1	PP2NIYLKPKKAGGMKITFQTPPKNGLDEKMVMNILRDYKILNCEVLVRDENV~TVDDFI
FG05139.1 	PP2NIYLKVKTAGGMKITFSSPPKR-LDEKMLYNILRDYKILNCEVLVRDENA~TVDDFI
AN2244.1  	PP2NIYLKAKKAGGMKITFQTPPKY-LDEKMIYNVLRDYKMLNCEVLVRDENA1TIDDFI
          	** ***** *.******.* :***  *****: *:*****:**.***:**:*. *:**:*

MG08640.1 	DVIMKDHRKYIK0C~LYVYNKIDSVSLDFLDKLAREPHTVVMSCELDLGIQDVIDRCWKE
NCU01183.1	DVIMKDHRKYIK~C~LYVYNKIDSVSLDFLDKLAREPYTCVMSCELDLGIQDVIDRCWKE
FG05139.1 	DVIMKDHRKYIK~C~LYVYNKIDSVSLDFLDQLAREDHTVVMSCEDDLGIQDVIDRCWKE
AN2244.1  	DVIMKDHRKYIP~C2LYVYNKIDSVSIEFLDQLAREPHTAVMSCELDLAVQDVVERIWKE
          	***********  * ***********::***:**** :* ***** **.:***::* ***

MG08640.1 	LSLVRIYTKR~KGIDPDFSEALIVRSNSTIEDVCDRIHRTLKETFKYALVWGASARHVPQ
NCU01183.1	LKLIRIYTKR~KGTDPDFSEALIVRSNSTIEDVCDRIHRTLKETFKYALVWGASARHIPQ
FG05139.1 	LKLIRIYTKR~KGAEPDFGEALIVRSNSTIEDVCDRIHRTLKDTFKYAMVWGASARHIPQ
AN2244.1  	LRLMRIYTKR2KGVEPDFSEALIVRNNSTIEDVCDQIHRTLKETFKYALVWGASARHIPQ
          	* *:****** ** :***.******.*********:******:*****:********:**

MG08640.1 	RVGLGHPVADEDVVYIVSGYKA
NCU01183.1	RVGLGHPVADEDVVYIVSGWKA
FG05139.1 	RVGLSHPVADEDVVYICTAWKA
AN2244.1  	RVGLSHVVADEDVVSIVAK---
          	****.* ******* * :
```
